# The New Tuberculosis Specialist

**Published:** 1913-06-21

**Authors:** 


### The Hospital

The Workers' Newspaper of Administrative Medicine and Institutional Life, Administration, National Insurance and Health.

No. 1408, Vol. LIV. SATURDAY, JUNE 21, 1913. PRICE ONE PENNY.

### THE NEW TUBERCULOSIS SPECIALIST.

Some time ago Sir Clifford Allbutt put forward, and we commented upon, a proposal to do away with the existing dichotomy of medical and surgical clinical practice, and to encourage instead a polychotomy which should correspond to the various regions of the human body. On his view, a man with an abdominal ailment should not need to go to a physician or a surgeon according as to whether the trouble were diarrhœa or appendicitis, but should be able to find a specialist in diseases of the region affected who would undertake the case, and see it through from start to finish, whatever its nature, and whatever the developments. This, of course, amounted to not much more than bidding God-speed to the present tendency to specialism. Gynæcologists are surgeons as well as physicians; so are ophthalmologists, so are practitioners in "nose and throat." In these three chief instances the ultimate motive for specialisation has been the same as that put forward by the Cambridge Professor of Medicine—namely, the benefit of the patient; because the patient stands to gain by having available the advice of one exclusively occupied with one section of diseases. But although the motive for specialisation had to do with the laity, the initiative in establishing it had nothing to do with them. The initiative in forsaking general for these forms of special practice was taken by medical men themselves.

But before the time of Bowman, or of Spencer Wells, or of Morell Mackenzie, medical specialisation had begun in other forms and under other auspices. And here the initiative was not due, directly at any rate, to the medical profession, but to laymen. The motive was partly the same—namely, the good of the sick; but a still greater one was the needs, the convenience, and not least, the pocket, of the community. It being obvious that lunatics and fever patients must be segregated, it was clearly indicated to make it one doctor's special business to look after each variety. When hygiene had proved itself, the want became apparent for specialists in preventive medicine; and as their subject, never homogeneous, became more and more heterogeneous, the medical officer of health had to be given departmental assistants, such as education medical officers. The next development provides the topic of present remark. A few words seem to be called for concerning the now familiar "tuberculosis officer."

It will be interesting to see what professional position this individual attains in the next decade or so. On the one hand, he has that primary requisite to special competence—namely, abundant special clinical material. Already, in any of the great towns, every consumptive is seen by the tuberculosis officer, and a large proportion of them also treated by him. He thus comes into more intimate contact with the disease than did the consultant, who saw private cases at long intervals (and often, as is now realised, built up a top-heavy structure of prognosis and treatment upon the poor foundation of auscultatory evidence) and merely visited hospital patients. He will, we think, be encouraged to assume somewhat the position of an authority, for town councils are democratic bodies, socially and temperamentally rather out of touch with Harley Street, and will prefer to be guided by an employee's report rather than by the *ex-cathedrâ* deliverances of a comparative alien. By now, too, there is a good deal in the therapeutics of consumption beyond the compass of a middle-aged consultant clinician. There is intensive tuberculin treatment—the tuberculosis officer can soon get more experience in that than anyone else in the neighbourhood, saving the sanatorium superintendent. There are all the applications of the *Lungenkollapstheorie* recently noticed in these columns; and in his hospital or annexe for advanced cases the tuberculosis officer can quite well practise artificial pneumothorax treatment and even extra-pleural thoracoplasty. Many of these advanced cases will, too, have laryngeal tuberculosis, the care of which has always been left by the heretofore authority on consumption to laryngologists. It cannot but be an advantage to the community to have a medical man so completely equipped to deal with one of the commonest of serious diseases.

No doubt about the immediate advantage. The question is, however, will research and the highest clinical excellence be similarly furthered? Let us not forget, despite the admitted competence of alienists and of fever hospital residents, that the fine flower of clinicians are raised on the old plan of competition for consultant status, and of specialisation according to certain regions of the human body. Tuberculosis is a wide-ranging disease which cuts across this last form of specialisation; and anyone who wishes to become, to use an esoteric colloquialism, a "tuberculosis man," just as others are "eye men" or "ear men," will have a different and more difficult task. A few there are who come up to the ideal specification, but how many who do not! The fact is, as hinted already from one or two quarters, the local bodies charged to provide themselves with these medical officers are not really fit as yet for the responsibility of election. In many cases to date local influence has shown itself to be far the best qualification for success. This is one of the initial false steps this interesting new section of the medical profession will have to retrieve. The lines it should adopt in endeavouring to do so are, we believe, those indicated above.

